# Short communication: Binocular rivalry dynamics during locomotion

**DOI:** 10.1371/journal.pone.0300222

**Published:** 2024-04-01

**Authors:** Brian Szekely, Robert Keys, Paul MacNeilage, David Alais

**Affiliations:** 1 Department of Psychology, University of Nevada, Reno, Reno, Nevada, United States of America; 2 School of Psychology, University of Sydney, Sydney, New South Wales, Australia; Istituto di Ricerca e di Studi in Ottica e Optometria, ITALY

## Abstract

Locomotion has been shown to impact aspects of visual processing in both humans and animal models. In the current study, we assess the impact of locomotion on the dynamics of binocular rivalry. We presented orthogonal gratings, one contrast-modulating at 0.8 Hz (matching average step frequency) and the other at 3.2 Hz, to participants using a virtual reality headset. We compared two conditions: stationary and walking. We continuously monitored participants’ foot position using tracking devices to measure the step cycle. During the walking condition, participants viewed the rivaling gratings for 60-second trials while walking on a circular path in a virtual reality environment. During the stationary condition, observers viewed the same stimuli and environment while standing still. The task was to continuously indicate the dominant percept via button press using handheld controllers. We found no significant differences between walking and standing for normalized dominance duration distributions, mean normalized dominance distributions, mean alternation rates, or mean fitted frequencies. Although our findings do not align with prior research highlighting distinctions in normalized dominance distributions between walking and standing, our study contributes unique evidence indicating that alternation rates vary across the step cycle. Specifically, we observed that the number of alternations is at its lowest during toe-off phases and reaches its peak at heel strike. This novel insight enhances our understanding of the dynamic nature of alternation patterns throughout the step cycle.

## Introduction

Binocular rivalry is a phenomenon in which visual perception oscillates due to conflicting stimuli being presented monocularly to each eye [1]. Research suggests that the purpose of binocular rivalry is to resolve the diplopia that is caused by naturally occurring discrepancies in the images at each eye [2]. In addition, it is suggested that binocular rivalry is governed by intrinsic oscillators that control how often an action potential occurs [3].

Binocular rivalry can be impacted by many factors, including retinal image motion and non-visual sensory signals [4–7]. Given that human locomotion under natural conditions drives retinal image motion as well as non-visual (i.e. vestibular) stimulation, it is possible that binocular rivalry is modulated by locomotion. While prior research has concluded that walking does not change the fundamental nature of binocular rivalry [4], this previous study did not investigate how the temporal frequency of the rivaling stimuli or walking speed affect binocular rivalry.

Given that alternation rates in binocular rivalry are affected by temporal changes in stimuli [8], and given that the human locomotion step rate has a resonant temporal frequency of approximately 2 Hz [9], binocular rivalry during locomotion might exhibit modulations at this frequency. Furthermore, there are other reasons to expect that locomotor rate may affect binocular rivalry rates. Prior research has observed that walking speed may affect eye movements, specifically saccadic rates [10], as well as alpha wave power, which correlates with cognitive performance in some tasks [11]. Therefore, there are several possible mechanisms by which rivalry alternation and dominance rates could be modulated by locomotor phase.

Building on prior research, the current study aims to investigate the impact of locomotion and locomotor phase on binocular rivalry, using orthogonal sine gratings with drift rates that align with frequency harmonics of human locomotion. We hypothesized that measures of binocular rivalry would be impacted by the act of locomotion.

## Materials and methods

### Participants

Six human subjects (4 female, 2 male) ranging in age from 23 to 28 years old (25 ± 3) participated in the experiment. Participants were recruited between November 2019 to January 2020. All participants had normal or corrected-to-normal vision, and all reported some prior experience with VR. The experimental procedures were approved by the University of Sydney Human Research Ethics Committee (No. 2016/662), with written informed consent obtained prior to participation in the study. The authors kept information that could identify individual participants during and after data collection in a password-protected computer to ensure confidentiality.

### Materials

Stimuli were displayed using an HTC Vive Pro Eye head-mounted display (HMD), which featured a 110° diagonal FOV, a refresh rate of 90 Hz, and 2880 x 1600 pixel resolution. This HMD was also used to collect eye tracking data at a rate of 30 Hz. Subjects responded to prompts in the experiment using a HTC Vive controller. Gait kinematics were tracked with a HTC VIVE Tracker secured to the participants’ ankle.

### Procedure

**Fig 1:**
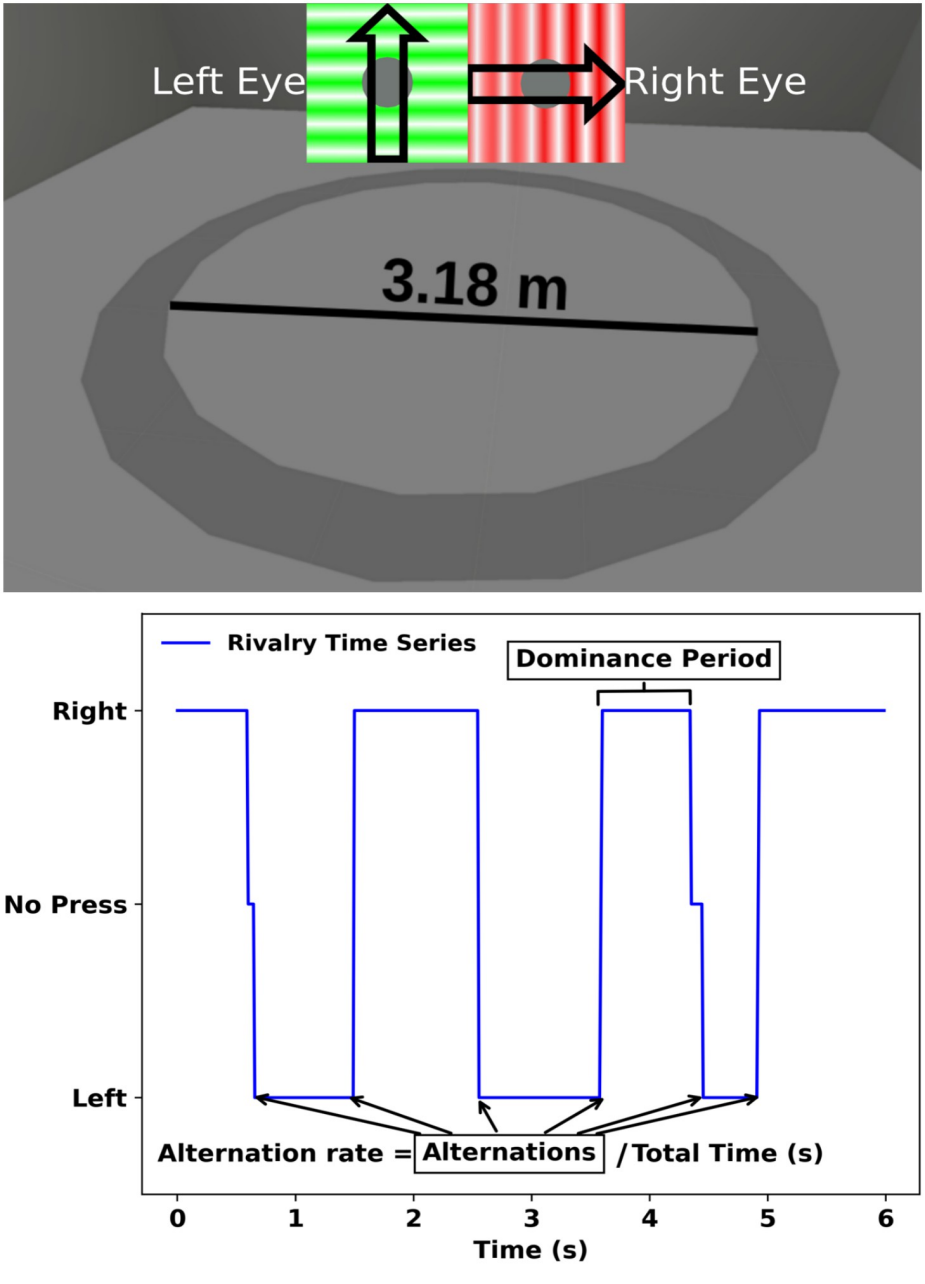
Experimental setup and example data analysis. The top panel shows the virtual visual environment with the path that the participants walked, as well as the green and red drifting sinusoidal gratings. The arrows on the gratings indicate the drift direction. The bottom left panel shows example response data; the blue trace indicates button presses over time, while the green trace illustrates the best-fitting sine wave. Dominance period and alternation rate are also depicted. The bottom right panel shows example goodness-of-fit analysis for a single subject and condition.

Subjects completed a total of 2 blocks (walking or standing) of 8 conditions consisting of counterbalanced paired colors, temporal frequencies, and drift directions of a 1 cycle-per-degree (cpd) grating which spanned 6° of visual angle (Fig 1). The task was to continuously track alternations in rivalry dominance between the red and green gratings by holding down the appropriate button on the controller when one color was dominant. Each condition lasted 60 seconds, resulting in a total exposure time of 8 minutes per block. Subjects were allowed to take a break of 5 minutes before completing the second block. In the walking block, subjects walked the circumference of a 3.18 m diameter (10 m circumference) circle in virtual space. In the standing block, subjects completed the same task while standing still. These blocks were counterbalanced, with half of the subjects completing the standing block first, and half completing the walking block first. The colors used for the grating were chosen because they are known to elicit binocular rivalry and are easily distinguishable [12, 13]. Temporal frequency of the grating was set to be either 0.8 Hz or 3.2 Hz to match the harmonic frequency of human locomotion [9]. Previous research has demonstrated that dynamic stimuli, such as moving patterns or gratings, can impact the alternation rate and dominance duration in binocular rivalry, resulting in more robust measurements [14]. The temporal frequencies were counterbalanced: one eye was presented with a frequency of 0.8 Hz, while the other eye was presented with a frequency of 3.2 Hz. Drift direction of the grating was set to drift horizontally leftward or vertically upward.

### Data analysis

The main dependent measures were alternation rate and perceptual dominance duration (Fig 1). All metrics were calculated separately for standing and walking. Alternation rates were calculated as the number of perceptual switches (i.e., left trigger release followed by a right trigger hold, and vice versa) divided by the total time across the 8 conditions (480 seconds; [15, 16]). Mixed percepts were collected when participants did not press either trigger. We analyzed the alternate rates not only between walking and standing, but also as a function of the step cycle. The step cycle traditionally denotes the duration from the heel strike of one leg to the heel strike of the opposite leg [17]. The step cycle was calculated from the HTC Vive HMD, with the start of the step cycle (0%) being when the head is at it highest vertical position (toe-off phase) to the next subsequent highest vertical position (100%). We then binned the alternations in 10% bins of the step cycle. Perceptual dominance duration was the average total time that a stimulus dominated the visual percept; in other words, the average duration that a trigger was held down, whether for the red or the green stimulus [18]. Dominance durations less than 250 ms were removed, as they were deemed too short to qualify as a valid dominance duration. The remaining dominance durations were then normalized by dividing each individual’s event by that person’s overall average from their walking and standing dominance events [4]. Previous research shows that dominance durations follow a gamma distribution [1]. Therefore, we fit a gamma distribution to the dominance duration, finding the best shape (λ) and scale (r) parameters. The shape parameter controls the form of the distribution and the scale parameter governs the spread of the distribution.

Walking speed was calculated for each step from the head tracking data by first identifying the beginning and end of a step using the peak linear y-positions (or heave) at each step. Speed was then computed as the forward distance covered in each step divided by the step duration. Speed across all steps was then averaged. Differences between walking and standing data were evaluated using a one sampled paired t-test. To compare each 10% step cycle window with one another, we used two-sample paired t-tests, adjusting for multiple comparisons using a Bonferroni correction. To evaluate statistical robustness, power analyses were conducted. Cohen’s d was utilized to compute the effect size based on means and standard deviations from the conditions (Stand vs. Walk) across dependent variables. Using a significance level of 0.05, the power analysis indicated an achieved power of approximately 0.05 for assessing alternations between standing and walking. For the assessment of dominance duration, an achieved power of approximately 0.12 was observed. To achieve a power of 0.8, a minimum detectable effect size of approximately 1.80 Cohen’s d would be needed given the current sample size. Distributions of dominance durations were compared using the Kolmogorov–Smirnov test.

## Results

**Fig 2:**
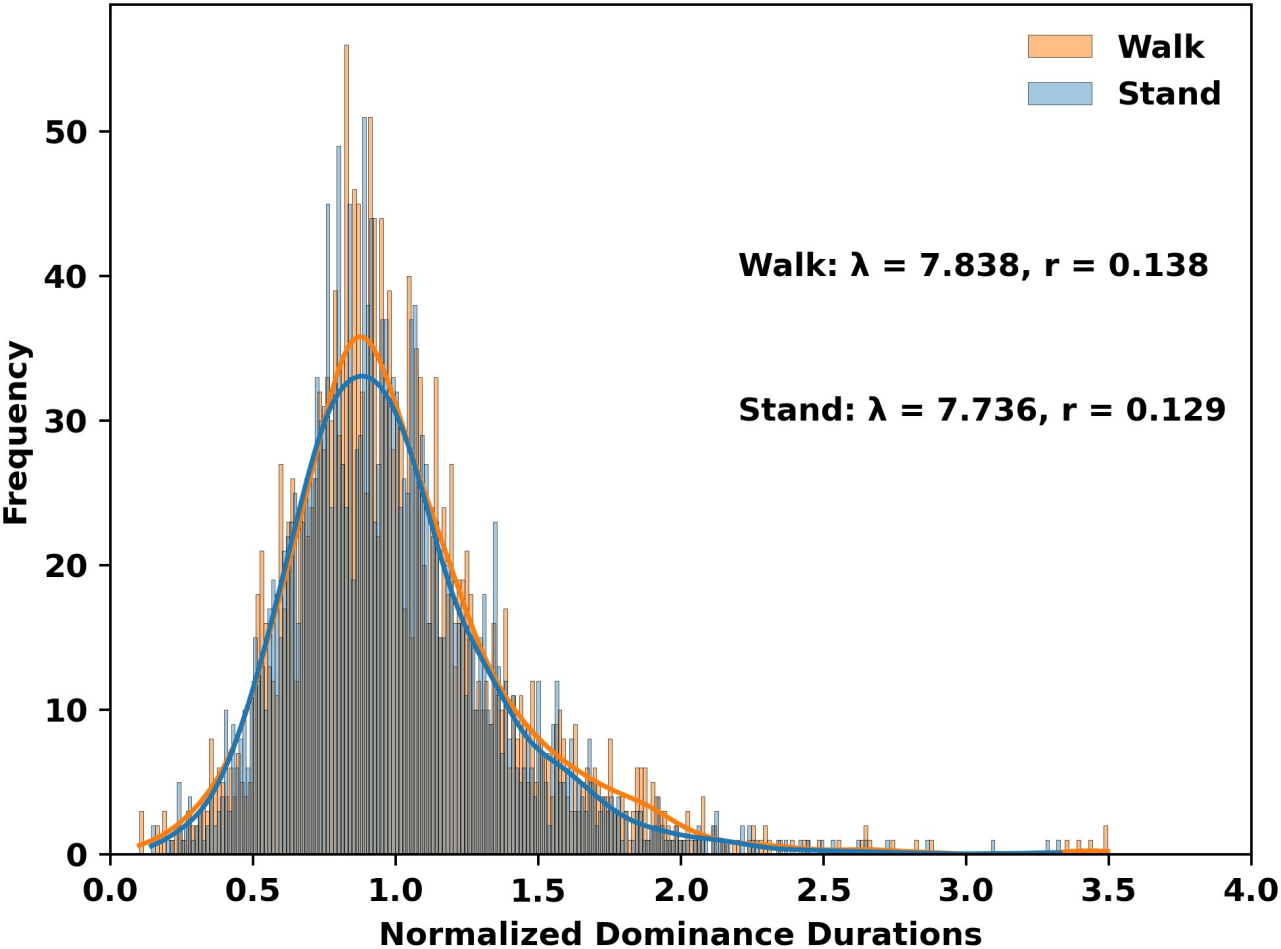
Dominance duration distributions between standing and walking conditions (p = 0.26). Data are means ± standard deviations. The overlaid lines are kernel density estimates. Lambda and r are the shape and scale parameters from the fitted gamma distribution, respectively.

**Fig 3:**
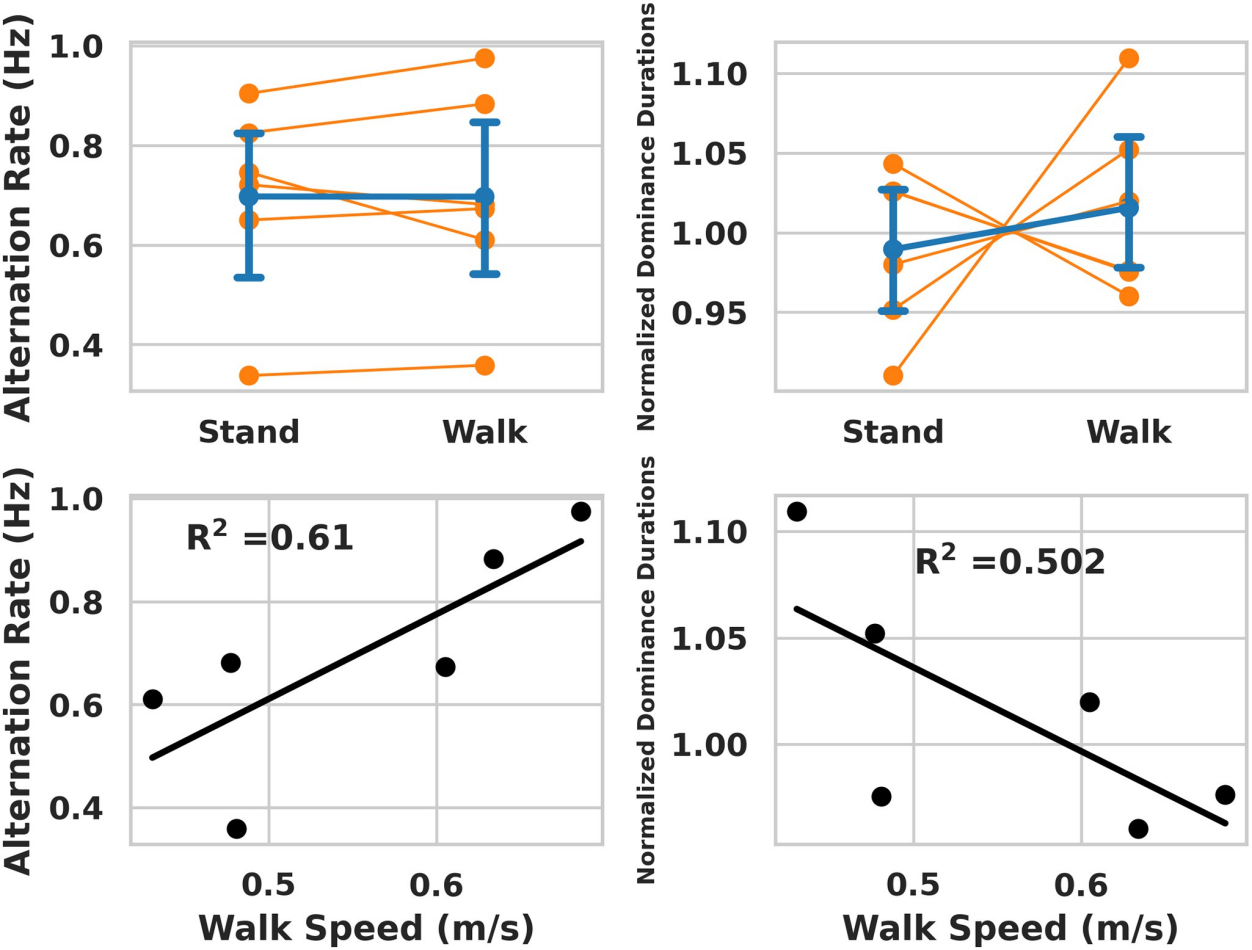
Alternation rate and dominance durations per condition, and also as a function of walking speed. Bar plots (top) show means ± standard deviations for alternation rate (p = 0.5) and dominance durations (p = 0.58). Scatter plots (bottom) show mean alternation rate (p = 0.06) and dominance durations (p = 0.30) as a function of walking speed in the walking condition.

To investigate differences in normalized dominance duration between walking and standing, we generated histograms across all conditions and participants (Fig 2). Both of these histograms exhibited a gamma distribution, a pattern previously observed in dominance duration distributions [19]. These distributions for walking (N = 2007) and standing (N = 1996) were not significantly different (D = 0.031, d = 0.06,p = 0.294). Furthermore, the average normalized dominance duration was found to be equivalent between walking and standing (Fig 3;t(5) = -0.587, d = 0.48, p = 0.58).

**Fig 4:**
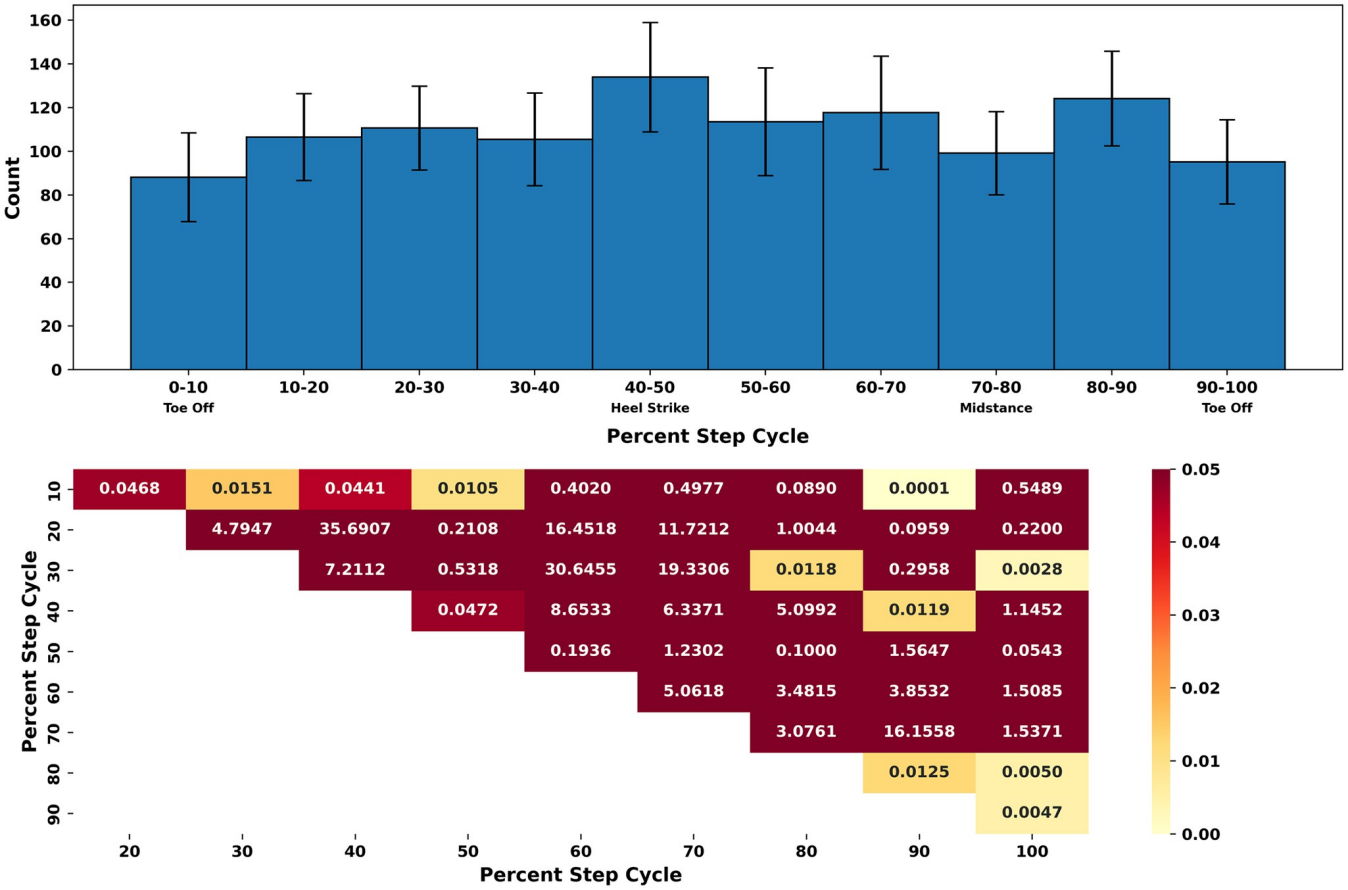
The alternation rate varies in relation to the step cycle. The step cycle is initiated from the toe-off phase of one foot until the toe-off of the other foot. The top plot displays the average number of alternations per 10% bin, with error bars indicating the standard deviation. The bottom plot illustrates the comparison between all 10% bins, displaying their respective p-values. The color intensity corresponds to the p-values, with darker red indicating higher p-values, and the lighter yellow indicating a lower p-value. The number of alternations from 0-10% were significantly lower compared to the alternations from 10-50% after the Bonferroni correction was applied *t*(10) = (−9.36, −6.86), *p* < 0.05). Furthermore, The number of alternations at 80-90% were statistically greater than 0-10% (*t*(10) = 23.23, *p* < 0.05). 20-30% alternations were significantly greater than alternations at 70-80% (*t*(10) = 9.14, *p* < 0.05) and alternations at 90-100% (*t*(10) = 12.31, *p* < 0.05). 30-40% alternations were significantly lower than alternations at 40-50% (*t*(10) = −6.79, *p* < 0.05) and 80-90% (*t*(10) = −9.13, *p* < 0.05). 80-90% alternations were significantly greater than alternations at 70-80% (*t*(10) = 9.02, *p* < 0.05) and 80-90% (*t*(10) = 11.06, *p* < 0.05).

Alternation rate seemed to conform more closely to the slow temporal frequency that the participants viewed (0.8 Hz), rather than the faster temporal frequency (3.2 Hz; Fig 3) for both standing (0.73 ± 0.18 Hz) and walking (0.68 ± 0.20 Hz). Alternation rates did not differ between walking and standing (t(5) = -0.0111, d = 0.002, p = 0.5) and no participant had an alternation rate above 1 Hz. The alternation rate throughout the step cycle indicates that heel strike phase (40-50%) corresponds to the bins with the highest alternations across the entire step cycle. Conversely, the alternations during the toe-off phases has the lowest amount across the step cycle (Fig 4). Fig 4 provides a comprehensive presentation of statistical comparisons for each bin of the step cycle, along with their respective p-values.

We also analyzed the relationship between all dependent measures and walking speed (Fig 3, bottom). Alternation rates (*R*^2^ = 0.61; *p* = 0.06) trended towards positive associations with walking speed, while normalized dominance duration trended towards a negative association (*R*^2^ = 0.50; *p* = 0.11). However, none of the correlations were significant. These correlations lack appropriate statistical power, and should be interpreted as pilot data.

**Fig 5:**
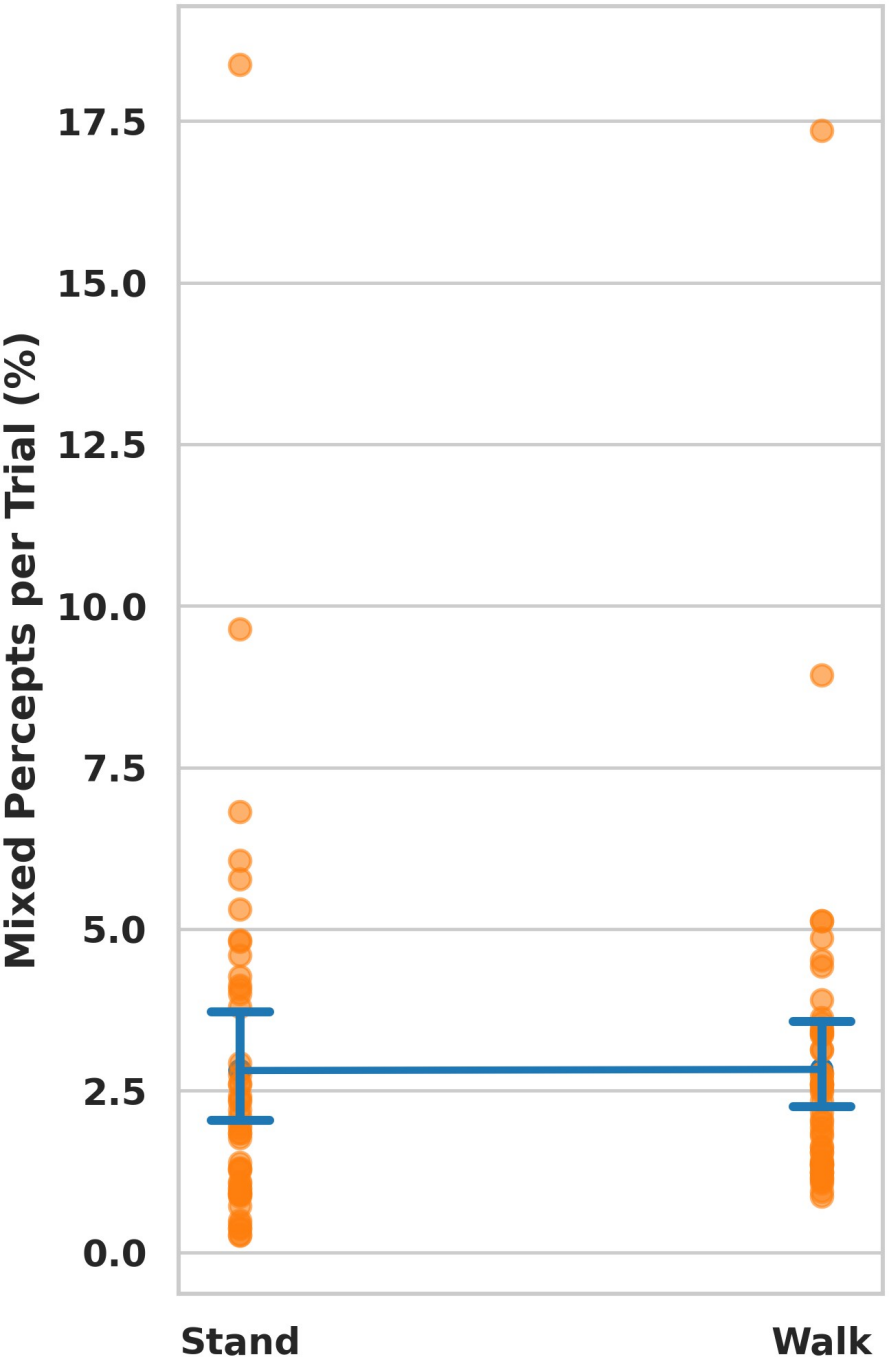
The data show the total duration, in seconds, of mixed percepts encountered by participants in both standing and walking conditions. The blue line illustrates the mean and standard deviation, with orange dots indicating mixed percept data for individual trials.

To evaluate the prevalence of mixed percepts among participants, we recorded the duration during each trial—both for standing and walking—when individuals refrained from holding either trigger (Fig 5. Our analysis revealed that, on average, participants encountered mixed percepts for 2.83 ± 2.52% of the total trial time during walking and 2.81 ± 3.01% for standing. This roughly translates to 1-4 seconds of mixed percepts per trial, a relatively small proportion given the 60-second duration of each trial.

## Discussion

The purpose of this study was to investigate the influence of locomotion on binocular rivalry, and to evaluate the alternation rate over the step cycle. Our hypotheses were that locomotion would affect binocular rivalry, such that the alternation rate would follow the 2Hz frequency of walking.

Comparing dominance duration between walking and standing, we found that the normalized distributions (Fig 2) were not significantly different. In addition, no significant differences were observed for the alternation rate or normalized dominance duration, (Fig 3, top). Our results contrast with previous studies that reported that normalized dominance distributions are significantly longer during walking than during sitting [4]. One possible explanation for the discrepancies between this study and previous research may be the control condition used, which was sitting in the previous study versus standing in the present study. Prior research suggests that standing enhances cognitive control when compared to sitting [20]. While binocular rivalry is thought to be mainly driven by low-level inhibitory control, high-level executive control may influence dominance durations [21]. Thus, it is plausible that previously observed effects of walking is due to the use of sitting versus standing as the control condition.

While the present study does not show an overall effect of walking itself on the duration of ambiguous stimuli during visual processing, results suggest that walking speed may impact binocular rivalry. There was a near significant positive correlation between each individual’s mean walking speed and their alternation rate. This suggests that individuals who walk faster may also have a faster alternation rate in binocular rivalry. In other words, this suggests that an individual’s walking speed could help determine the frequency of changes in binocular rivalry. We also examined whether walking speed was correlated with other binocular rivalry metrics, but these correlations failed to reach significance. These results exhibit insufficient statistical power. Future research with improved statistical power is needed to evaluate the validity of this relationship.

The alternation rate observed throughout the step cycle reveal a discernible bias in responses towards specific phases, notably the heel strike (40-50%). Conversely, the toe-off periods exhibit the lowest alternations among all phases. This suggests a potential periodicity in the alternation rate during locomotion. The vestibulo-ocular reflex demonstrates heightened activity during specific phases of the gait cycle [22]. Additionally, Reaching error have been shown to oscillate as a function of the step cycle [23]. However, the connection between locomotion and perception has not been established. The observed oscillations in visual perception suggest the possibility that the rhythmic nature of locomotion could induce rhythmic patterns in visual perception.

The near-significant correlation between walking speed and alternation rate is the first suggestion in the literature that binocular rivalry may be impacted by walking speed, indicating a potential impact of motor behavior on visual perception Prior research has reported that retinal image motion could cause binocular rivalry alternations [24]. It is possible that those with higher walking speeds may have had greater retinal image motion, which could have led to shorter dominance periods and increased alternation rates. However, it is noteworthy that the present study did not directly measure retinal image velocities. These analyses did not have sufficient statistical power and should be interpreted with caution. Further investigations are needed to confirm whether retinal image motion during locomotion impacts dominance periods and alternation rate.

The authors acknowledge that this study has limitations. First, this study had fewer participants than earlier studies on binocular rivalry and motor behavior [4, 7]. The relatively small sample size may have constrained our ability to detect smaller effects. For example, our power analysis suggested that our sample size would have only allowed for the detection of a very large effect (Cohen’s d of 1.80). This implies that walking may either not have a significant effect on binocular rivalry, or that, if present, the effect may be smaller in magnitude. Thus, caution is warranted when interpreting these results within the context of sample size limitations. Another limitation stems from the fact that participants were not explicitly instructed to report mixed percepts. Mixed percepts analyzed in Fig 5 were computed as times when participants did not have a button pushed. However, the lack of explicit instructions for reporting mixed percepts may have led participants to bias their responses towards reporting dominant percepts, even in cases where the dominance of either eye was only partial. This is particularly plausible given the large size of the stimuli. Such bias could have compromised the reliability of rivalry measurement compared to previous studies, consequently reducing the measured effect size. However, it’s important to consider that the majority of rivalry studies typically don’t offer a third option; While it’s true that this setup might force participants to prefer one eye’s dominance when they might have experienced a mixed percept, since there are only two alternating percepts, this bias would likely affect both options similarly and not skew the data towards one or the other. In essence, using only two buttons isn’t a critical issue and wouldn’t systematically impact the data. Furthermore, it’s worth noting that walking could influence perceptual switches indirectly, potentially through retinal motion effects. This influence could occur either physiologically, due to a differential distribution of eye movements across the gait cycle, or due to an artifactual shift of the VR headset with every step. This study was exploratory in nature and should serve as pilot data for future research into the dynamics of visual perception during locomotion. Secondly, to ensure a standardized distance and height from the observer’s eyes, the stimuli were maintained at a fixed position relative to the head position. It is plausible that this methodological approach, which is arguably unnatural, could have influenced the functionality of the vestibular-ocular reflex, and thereby influenced binocular rivalry. Lastly, we used a circle for our walking paradigm; however, this may affect the vestibular signal. Future research could investigate how differing walking paths (e.g. straight versus circular) affect visual function.

## Conclusion

In conclusion, this study demonstrates that the act of walking may not have a significant impact on the dynamics of binocular rivalry. However, it does reveal that the dynamics of binocular rivalry may oscillate over the step cycle. Our findings suggest that the phase of the step cycle may influence the alternation rate. Further research is needed to examine the relationship between gait and basic visual function.
